# Protandim Protects Oligodendrocytes against an Oxidative Insult

**DOI:** 10.3390/antiox5030030

**Published:** 2016-09-07

**Authors:** Jamie L. Lim, Susanne M. A. van der Pol, Wia Baron, Joe M. McCord, Helga E. de Vries, Jack van Horssen

**Affiliations:** 1 Department of Molecular Cell Biology and Immunology, VU University Medical Center, Neuroscience Campus Amsterdam, 1081 HZ Amsterdam, the Netherlands; jamielim85@gmail.com (J.L.L.); sma.vanderpol@vumc.nl (S.M.A.P.); he.devries@vumc.nl (H.E.V.); 2 Department of Cell Biology, University Medical Center Groningen, University of Groningen, 9700 RB Groningen, the Netherlands; w.baron@umcg.nl; 3 Department of Medicine, Division of Pulmonary Science and Critical Care Medicine, University of Colorado at Denver, Aurora, CO 80045, USA; joe.mccord@ucdenver.edu

**Keywords:** multiple sclerosis, reactive oxygen species, Nrf2, antioxidant enzymes

## Abstract

Oligodendrocyte damage and loss are key features of multiple sclerosis (MS) pathology. Oligodendrocytes appear to be particularly vulnerable to reactive oxygen species (ROS) and cytokines, such as tumor necrosis factor-α (TNF), which induce cell death and prevent the differentiation of oligodendrocyte progenitor cells (OPCs). Here, we investigated the efficacy of sulforaphane (SFN), monomethyl fumarate (MMF) and Protandim to induce Nrf2-regulated antioxidant enzyme expression, and protect oligodendrocytes against ROS-induced cell death and ROS-and TNF-mediated inhibition of OPC differentiation. OLN-93 cells and primary rat oligodendrocytes were treated with SFN, MMF or Protandim resulting in significant induction of Nrf2-driven (antioxidant) proteins heme oygenase-1, nicotinamide adenine dinucleotide phosphate (NADPH): quinone oxidoreductase-1 and p62/SQSTM1, as analysed by Western blotting. After incubation with the compounds, oligodendrocytes were exposed to hydrogen peroxide. Protandim most potently promoted oligodendrocyte cell survival as measured by live/death viability assay. Moreover, OPCs were treated with Protandim or vehicle control prior to exposing them to TNF or hydrogen peroxide for five days, which inhibited OPC differentiation. Protandim significantly promoted OPC differentiation under influence of ROS, but not TNF. Protandim, a combination of five herbal ingredients, potently induces antioxidants in oligodendrocytes and is able to protect oligodendrocytes against oxidative stress by preventing ROS-induced cell death and promoting OPC differentiation.

## 1. Introduction

Multiple sclerosis (MS) is a chronic inflammatory disease of the central nervous system (CNS). Histopathological hallmarks include focal demyelinated lesions characterized by oligodendrocyte (OL) cell death, axonal damage, gliosis, microglial activation, and infiltration of lymphocytes and macrophages [1,2]. In chronic demyelinating lesions in advanced stages of the disease, OL loss is extensive and widespread [3,4,5]. As oligodendrocyte progenitor cells (OPCs) were detected in chronic lesions [5,6], it appears that premyelinating OLs fail to differentiate and ensheath the axon with myelin in chronic MS. Evidence is emerging that both pro-inflammatory cytokines and oxidative stress play a key role in OL cell pathology in MS.

Over the past decade, studies have shown that ROS-induced oxidative damage to cells in the CNS significantly contribute to demyelination and neurodegeneration in MS [7,8,9]. Markers of lipid and DNA oxidative damage have been detected in several CNS cells, including neurons, axons, myelin, and OLs in early active lesions with the highest levels of expression in cells that morphologically resembled apoptotic OLs [10,11]. Furthermore, expression of genes involved in ROS production, in particular the subunits of ROS-synthesizing enzyme nicotinamide adenine dinucleotide phosphate (NADPH) oxidase (NOX), were profoundly upregulated in cases of acute MS, specifically in activated microglia and macrophages in close vicinity of OLs [12].

In vitro studies demonstrated enhanced susceptibility of OLs to ROS-induced oxidative damage and cell death compared to astrocytes [13,14,15] and microglia [16,17]. OPCs are even more vulnerable than mature OLs to oxidative stress in vitro [18,19,20,21,22]. Factors contributing to the increased susceptibility of OLs to oxidative stress include the high intracellular iron content of the cells and relatively low levels of endogenous antioxidant proteins, which are lower in OPCs compared with mature OLs [14,15,23,24]. Furthermore, it was demonstrated that ROS are able to block OL maturation in vitro by arresting them in the progenitor phase by decreasing the expression of genes involved in OL differentiation and increasing the expression of genes known to inhibit differentiation [25]. Not only ROS, but also cytokines, such as tumor necrosis factor-α (TNF), may contribute to OL pathology in MS. In several studies, TNF was shown to selectively damage OLs and myelin in vitro [26,27] and was toxic to OL cultures [28,29]. Furthermore, TNF inhibited the differentiation of OPCs into mature OLs [30,31]. Although the mechanisms and time frame of ROS- and cytokine-induced cell death may differ, it is conceivable that both factors contribute to OL pathology in MS. Thus, a therapeutic compound that is able to protect OLs from both ROS- and cytokine-induced damage would have clinical value in MS [32]. Activation of the nuclear factor erythroid-2 related factor-2 (Nrf2) pathway involves the transcription of multiple (antioxidant) proteins, including heme oxygenase 1 (HO-1) and NAD(P)H: quinone oxidoreductase 1 (NQO-1), which protect cells from ROS- and cytokine-induced damage and cell death. Nrf2-activating compounds, like sulforaphane (SFN) [33,34,35,36,37,38], fumaric acid esters [39,40,41,42], and Protandim^®^ [43,44,45] are known to induce antioxidant enzymes in various cell types, have cytoprotective properties and reduce clinical signs in the experimental autoimmune encephalomyelitis animal model for MS. The fumaric acid ester dimethylfumarate (DMF) (and its active metabolite monomethylfumarate (MMF), in particular, have shown efficacy in MS, as the DMF-containing oral formulation Tecfidera™ was successful in two phase 3 clinical trials and is now used in the clinic to treat relapsing-remitting MS patients.

Here, we explored the efficacy of these cytoprotective compounds in protecting OLs against an oxidative and inflammatory insult. Our data showed that Protandim, a phytochemical compound consisting of five herbal ingredients, robustly increased antioxidant protein production in primary rat OLs. Treatment of Protandim protected OLs against oxidative insults and counteracted ROS-induced inhibition of OPC differentiation. Altogether, the results indicate that Protandim may have therapeutic potential for protecting OLs against oxidative insult in MS.

## 2. Materials and Methods

### 2.1. Cell Cultures

OLN-93 cells, a cell line established from spontaneously transformed rat brain glial cultures [46], were cultured in Dulbecco’s modified Eagle’s medium (DMEM) supplemented with 10% fetal bovine serum (FBS, Hyclone, Logan, UT, USA), penicillin, and streptomycin at 37 °C. OLN93 cells were plated in 96-wells plates (25,000 cells/well; Greiner Bio-One, Frickenhausen, Germany) or 24-well plates (50,000 cells/well; Greiner Bio-One). Primary Oligodendrocytes. Primary OLs were isolated from 0-day-old to 2-day-old Sprague Dawley rats, as described previously [47]. Animal experiments were approved by the Animal Experiments Review Board of the VU University Medical Center. Isolated OPCs were plated in 96-well plates (25,000 cells/well; Greiner Bio-One), 24-well plates (50,000 cells/well; Greiner Bio-One) or ibidi μ-Slide 8 well plates (30,000 cells/well; Ibidi, Martinsried, Germany) pre-coated with poly-l-lysine (PLL, 5 µg/mL; Sigma-Aldrich, St. Louis, MO, USA) for functional, protein or immunocytochemical analysis, respectively. For the differentiation studies, OLs were synchronized to OPCs by culturing in SATO medium [47] supplemented with the growth factors bFGF-2 (10 ng/mL; Peprotech, Rocky Hill, NJ, USA) and PDGF-AA (10 ng/mL; Peprotech) for 2 days before the cells were treated. For the studies with mature OLs, cells were directly incubated in SATO medium supplemented with 0.5% fetal calf serum (FCS) and cultured for 7 days (Bodinco, Alkmaar, the Netherlands). The plated OL cultures were estimated to be ~90% pure; ~5% of cells were astrocytes and ~5% microglia.

### 2.2. Functional Analysis of Oligodendrocytes

Primary OLs were allowed to mature for 7 days in SATO medium supplemented with 0.5% FCS. The cells were then treated for 24 h with sulforaphane (Sigma-Aldrich) (5 µM), monomethyl fumarate (Sigma-Aldrich) (90 µM), Protandim (LifeVantage, Sandy, UT, USA) (60 µg/mL) or their respective vehicle control, dimethylsulfoxide (DMSO) or ethanol (EtOH), followed by 4 h treatment with tert-butyl hydrogen peroxide (Sigma-Aldrich) or glucose oxidase (1:750,000; Sigma-Aldrich), two different methods of ROS exposure. Cell viability after tert-butyl hydrogen peroxide and glucose oxidase treatment was assessed using the LIVE/DEAD viability/cytotoxicity kit (Invitrogen, Carlsbad, CA, USA) according to manufacturer′s protocol. Fluorescent signals of alive and dead cells were measured with a fluorometer (FLUOstar Galaxy, BMG Lab technologies, Offenburg, Germany) and the ratio between live and dead cells was calculated. To assess the protective function of Protandim on OPC maturation under the inhibitory influence of inflammation or oxidative stress, OPCs were synchronized by growth factors for 2 days and subsequently treated with 30 µg/mL of Protandim or EtOH, as vehicle control. After 24 h, the medium was removed and replaced by SATO medium without vehicle control or Protandim, supplemented with 0.5% FCS in the absence or presence of either 10 ng/mL TNF or 10 µM tert-butyl hydrogen peroxide for 5 days.

### 2.3. Immunocytochemistry

To ensure purity of OPC culture and to determine differentiation of OPCs under experimental conditions, cells grown on PLL-coated ibidi slides were processed for detection of OL-specific antigens. Briefly, cells fixed with 4% paraformaldehyde were blocked with 10% goat serum and 0.1% Triton-X in phosphate-buffered saline (PBS) at room temperature for 45 min, and thereafter incubated with primary antibody overnight at 4 °C. Cells were then washed with PBS and incubated with secondary antibody for 30 min. After washing, cells were incubated with 4,6-diamidino-2-phenylidole (DAPI, 10 mg/mL; Sigma-Aldrich, St. Louis, MO, USA) for 5 min, washed again and kept in PBS. Primary antibodies used for the recognition of OL antigens were MBP (1:200) and Olig2 antibody (1:200), followed by goat anti-rat IgG Alexa Fluor or goat anti-rabbit Alexa Fluor (see ”antibody characterization” below). All secondary antibodies were diluted 1:200 in PBS with 1% goat serum and 0.1% Triton-X (Sigma-Aldrich). Five random fields per ibidi well/coverslip were scanned using a 10x lens of a Leica fluorescence microscope (DM6000), and the total number of cells, the number of cells labeled with each antibody, and the total number of MBP^+^/Olig2^+^ cells or MBP^−^/Olig2^+^ cells per field were counted with ImageJ and compared. These determinations were performed on cultures from three separate preparations.

### 2.4. Western Blotting

Protein isolation from primary rat OLs was performed using Laemmli buffer. Western blotting was performed, as described earlier [48,49]. Proteins were separated on 10% SDS-PAGE gels and transferred to polyvinylidene difluoride (PVDF) membranes (Bio-Rad Laboratories, Berkeley, CA, USA). After blocking in Odyssey blocking buffer (LI-COR Biosciences, Lincoln, AK, USA), membranes were incubated with primary antibodies overnight in Odyssey blocking buffer at 4 °C. Primary antibodies were detected by incubation with appropriate IRDye secondary antibodies (LI-COR Biosciences) for 1 h at room temperature in Odyssey blocking buffer and quantified using the Odyssey infrared imaging system (LI-COR Biosciences). Actin quantification was used to correct for total protein loading variation. Primary antibodies used were rabbit anti-HO-1 (1:1000), mouse anti-NQO-1 (1:1000), mouse anti-p62/SQSTM1 (1:1000) and mouse anti-actin (1:5000) (see “antibody characterization” below and in Supplementary Table S1).

### 2.5. Antibody Characterization

Primary antibodies used were rat anti-MBP (catalog no. MCA409S; RRID: AB_325004; Abd Serotec, Oxfordshire, UK), rabbit anti-Olig2 (catalog no. AB9610; RRID: AB_570666; Millipore, Billerica, MA, USA), rabbit anti-HO-1 (catalog no. ADI-OSA-150F; RRID: AB_1505620; Enzo Life Sciences, Farmingdale, NY, USA), mouse anti-NQO-1 (catalog no. ab28947; RRID: AB_881738; Abcam, Cambridge, UK), and mouse anti-p62/SQSTM1 (catalog no. ab56416; RRID: AB_945626), mouse anti-β-actin (catalog no. A5441; RRID: AB_476744; Sigma-Aldrich, St. Louis, MO, USA). The anti-MBP antibody was previously shown to stain myelin membrane formation in rat oligodendrocytes isolated from the brains of newborn pups using immunocytochemistry [50]. The anti-HO-1 antibody was demonstrated to stain a band at ~32 kDa in primary rat cerebellar neurons, whilst the anti-NQO-1 antibody was shown to stain NQO-1 at ~30 kDa human oral squamous cell carcinomas [51]. Rat pancreatic islet cells were stained by the anti-p62 antibody at approximately 62 kDa [52] and β-actin in mouse brain cortical cells was identified by the anti-β-actin antibody at a height of 42 kDa in a paper by Cheishvili et al. [53]. The secondary Alexa antibodies (goat anti-rat 488 (catalog no. A-11006; RRID: AB_10561520; Thermo Fischer Scientific, Waltham, MA, USA), goat anti-mouse 555 (catalog No.A-21428; RRID: 2535849; Thermo Fischer Scientific) were used at 1:200 dilution.

### 2.6. Glutathione Assay

Cells were plated at 25,000 per well in a 96-well plate (Greiner Bio-One) and allowed to mature for 7 days in SATO medium supplemented with 0.5% FCS. Following 24 h treatment with sulforaphane (5 μM), monomethyl fumarate (Sigma-Aldrich) (90 µM), Protandim (LifeVantage) (60 µg/mL), OLs were washed with PBS, and total glutathione was measured in a white 96-wells plate (Greiner Bio-One), using GSH-Glo, according to manufacturer’s instructions (Promega, Madison, WI, USA). Luminescence was measured with a FLUOstar Galaxy fluorometer.

### 2.7. Statistical Analysis

Results were analyzed using one-way analysis of variance (ANOVA) or student’s *t*-test for an average of 3 independent experiments. *p* < 0.05.

## 3. Results

### 3.1. SFN, MMF and Protandim Increase Antioxidant Enzymes and p62 Expression in OLN-93 Cells

**Figure 1:**
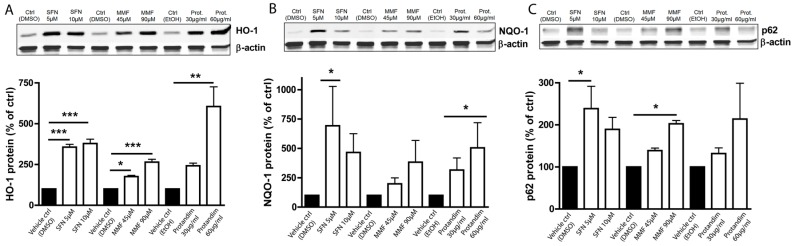
Nrf2-activators dose-dependently increase antioxidant protein expression in OLN-93 cells. HO-1 (**A**), NQO-1 (**B**) and p62 (**C**) protein expression levels after 24 h treatment in the OLN-93 oligodendrocyte cell line with 5 µM or 10 µM SFN, 45 µM or 90 µM MMF, 30 µg/mL or 60 µg/mL Protandim or their respective DMSO or EtOH vehicle control. Protein levels were assayed by Western blotting. Data are presented as percentage of control and expressed as the mean ± SEM of 3 independent experiments. All statistics reflect one-way ANOVA tests with post hoc Bonferroni correction; * *p* < 0.05, ** *p* < 0.01; *** *p* < 0.001.

OLN-93 cells most closely resemble 5-day to 10-day old (postnatal) cultured rat brain oligodendrocytes in terms of their morphological features and antigenic properties. To investigate and compare the efficacy of the Nrf2-activating compounds, and optimize compound concentrations for further experiments with primary rat oligodendrocytes, we first incubated OLN-93 cells with two different concentrations of SFN, MMF or Protandim or their respective vehicle control (DMSO or EtOH) for 24 h. The concentrations used were based on prior publications with the compounds [37,54,55] and dose-ranging experiments we performed in OLN-93 cells (data not shown). The concentrations were well-tolerated by the OLN-93 cells. MMF and Protandim dose-dependently increased the protein expression of HO-1 (Figure 1A) and NQO-1 (Figure 1B) compared to the highest concentration of their respective vehicle control. SFN, MMF and Protandim significantly increased HO-1 expression, whereas only SFN and Protandim significantly enhanced the expression of NQO-1. Furthermore, only SFN and MMF increased the expression of p62 in OLN-93 cells significantly (Figure 1C). Although 5 µM SFN appears to be optimal for both NQO-1 and p62 expression, a general dose-dependent trend can be observed with MMF and Protandim. Protein expression of HO-1, NQO-1 and p62 under EtOH- or DMSO-treated conditions were comparable to each other and untreated control.

### 3.2. SFN and Protandim Prevent ROS-Induced Cell Death of OLN-93 Cells

**Figure 2:**
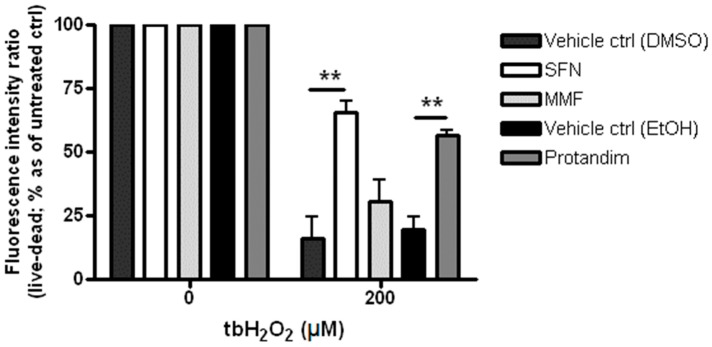
Sulforaphane and Protandim promote viability of OLN-93 cells under tert-butyl hydrogen peroxide-induced oxidative insult. OLN-93 cells were treated with 5 µM SFN, 90 µM MMF, 60 µg/mL Protandim or their respective DMSO or EtOH vehicle control for 24 h. After removal of medium, cells were subsequently exposed to either control medium or medium with 200 µM tert-butyl hydrogen peroxide. Cell viability was measured using an Invitrogen live/dead cytotoxicity kit. Data are presented as percentage of control and expressed as the mean ± SEM of 3 independent experiments. Statistics reflect one-way ANOVA test with post hoc Bonferroni correction; ** *p* < 0.01.

To elucidate the cytoprotective potential of SFN, MMF and Protandim to protect OLN-93 cells from oxidative stress, the cells were incubated for 24 h with either SFN (5 µM), MMF (90 µM), Protandim (60 µg/mL) or their respective vehicle control prior to exposing them to 200 µM tert-butyl hydrogen peroxide for 4 h. OLN-93 cell viability under EtOH- or DMSO-treated conditions were comparable to each other and untreated control. Furthermore, SFN and Protandim, but not MMF, significantly increased survival of OLN-93 cells (SFN: 65% ± 4.25% vs. 16% ± 8% of vehicle control; Protandim: 56% ± 1.7% vs. 19% ± 5% of vehicle control) (Figure 2).

### 3.3. SFN and Protandim Increase Antioxidant Enzymes and p62 Expression in Mature Primary Rat OLs

**Figure 3:**
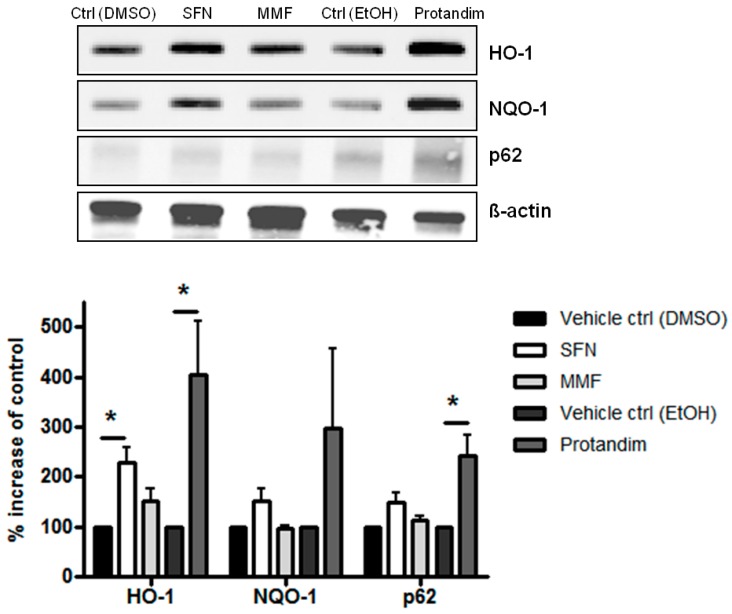
Nrf2-activators dose-dependently increase antioxidant protein expression in mature primary rat OLs. HO-1, NQO-1 and p62 protein expression levels after 24 h treatment in mature primary rat OLs, differentiated for 7 days, with 5 µM SFN, 90 µM MMF,30 µg/mL Protandim or their respective DMSO or EtOH vehicle control. Protein levels were assayed by Western blotting. Data are presented as percentage of control and expressed as the mean ± SEM of 3 independent experiments. Statistics reflect one-way ANOVA test with post hoc Bonferroni correction; * *p* < 0.05.

To investigate the potency of Nrf2-activating compounds in enhancing antioxidant proteins and p62 expression in mature primary rat OLs, we incubated mature rat OLs for 24 h with the most potent and tolerable concentrations of SFN, MMF or Protandim or their respective vehicle control, as determined by prior experiments with OLN-93 cells and viability assays with mature rat OLs. The concentrations used were 5 μM of SFN, 90 μM of MMF and 60 μg/mL of Protandim. Both SFN and Protandim significantly increased HO-1 protein expression in OLs, while NQO-1 and p62 protein levels were only significantly enhanced upon Protandim treatment. In contrast to OLN-93 cells, MMF treatment did not result in increased HO-1, NQO-1 and p62 protein expression (Figure 3). Furthermore, protein expression of HO-1, NQO-1 and p62 under EtOH- or DMSO-treated conditions were comparable to each other and untreated control.

### 3.4. Protandim Increases Glutathione Levels in Mature Primary Rat OLs and Protects OLs from ROS-Induced Cell Death

**Figure 4:**
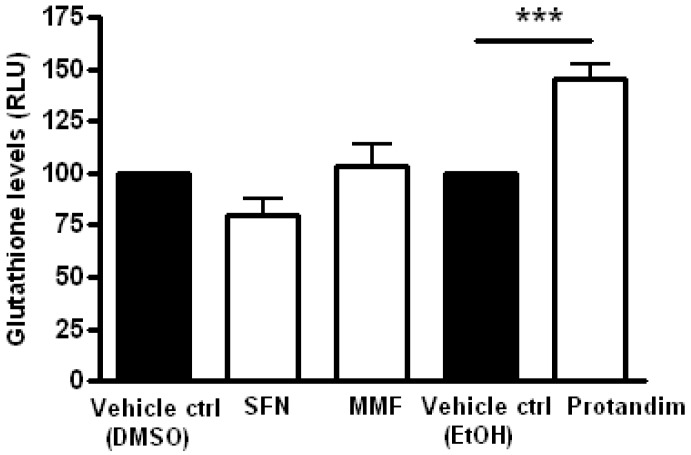
Protandim increases total glutathione levels in mature primary rat OLs. Mature primary rat OLs differentiated for 7 days were treated for 24 h with 5 µM SFN, 90 µM MMF,30 µg/mL Protandim or their respective DMSO or EtOH control. Total glutathione levels were assayed using the GSH-Glo kit from Promega. Data are presented as percentage of control and expressed as the mean ± SEM of 3 independent experiments. Statistics reflect one-way ANOVA test with post hoc Bonferroni correction; *** *p* < 0.001.

**Figure 5:**
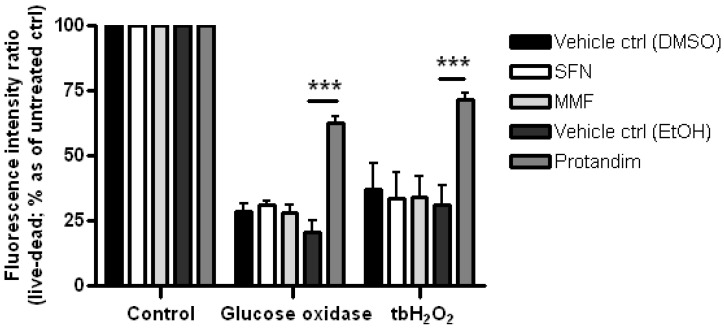
Protandim promotes viability of mature primary rat OLs under oxidative insult. Mature primary rat OLs differentiated for 7 days were treated for 24 h with 5 µM SFN, 90 µM MMF,30 µg/mL Protandim or their respective DMSO or EtOH vehicle control. After removal of medium, cells were subsequently exposed to either control medium or medium with 100 µM tert-butyl hydrogen peroxide (tbH_2_O_2_) or glucose oxidase (1:750,000). Cell viability was measured using an Invitrogen live/dead cytotoxicity kit. Data are presented as percentage of control and expressed as the mean ± SEM of 3 independent experiments. Statistics reflect one-way ANOVA test with post hoc Bonferroni correction; *** *p* < 0.05.

Glutathione is one of the most abundant mammalian intracellular thiol-containing antioxidants and represents a key buffer to maintain the cellular redox balance [56,57,58]. Hence, the capacity of the Nrf2-activators to enhance glutathione levels in mature OLs was investigated by incubating the cells for 24 h with SFN, MMF or Protandim or vehicle control. Protandim treatment significantly increased glutathione levels in primary OLs (Figure 4). Glutathione levels under EtOH- or DMSO-treated conditions were comparable to each other. To elucidate the cytoprotective potential of the Nrf2-activating compounds, primary rat OLs were incubated for 24 h with SFN, MMF, Protandim or vehicle control. Thereafter, the medium with compounds was removed and the cells exposed to an oxidative attack for 4 h. Only Protandim increased the percentage viability of primary rat OLs compared with vehicle control under both tert-butyl hydrogen peroxide exposure (Protandim: 71% ± 2% vs. 30% ± 7.5% of vehicle control) and glucose oxidase exposure (Protandim: 62% ± 2.5% vs. 20% ± 4.5% of vehicle control; Figure 5). OL viability under EtOH- or DMSO-treated conditions were comparable to each other and untreated control.

### 3.5. Protandim Promotes Differentiation of OPCs under Oxidative Conditions

**Figure 6:**
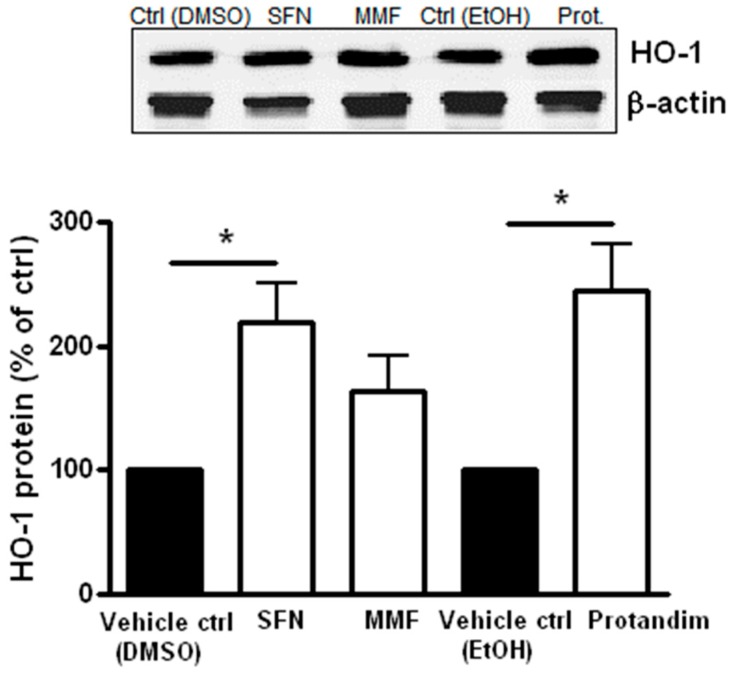
Nrf2-activators dose-dependently increase HO-1 protein expression in primary rat OPCs. After 2 day de-differentiation of OLs with growth factors bFGF-2 and PDGF-AA, primary rat OPCs were treated with 5 µM SFN, 90 µM MMF,30 µg/mL Protandim or their respective DMSO or EtOH vehicle control for 24 h. Protein levels were assayed by Western blotting. Data are presented as percentage of control and expressed as the mean ± SEM of 3 independent experiments. Statistics reflect one-way ANOVA test with post hoc Bonferroni correction; * *p* < 0.05.

**Figure 7:**
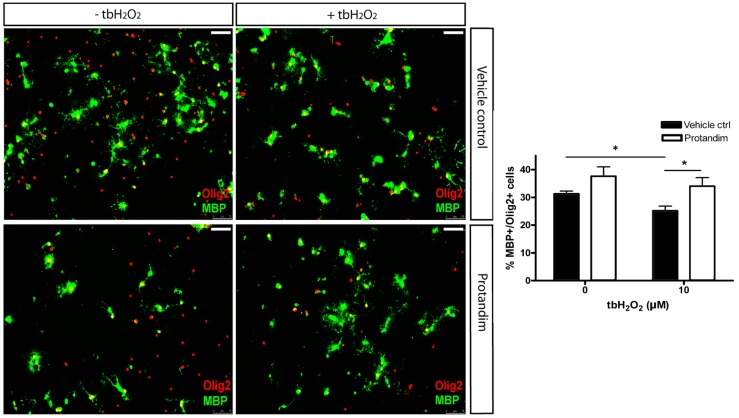
Protandim promotes differentiation of primary rat OPCs under oxidative stress. After 2 day de-differentiation of OLs with growth factors bFGF-2 and PDGF-AA, primary rat OPCs were incubated with 30 µg/mL Protandim or vehicle control (EtOH) for 24 h. After removal of medium, cells were subsequently exposed to either control medium or medium with 10 µM tert-butyl hydrogen peroxide (tbH_2_O_2_) for 5 days. MBP and Olig2 expression were assayed by immunocytochemistry. Data are presented as percentage of control and expressed as the mean ± SEM of 3 independent experiments. Statistics reflect student’s t-test, one-tailed; * *p* < 0.05.

In order to investigate the protective potential of SFN, MMF and Protandim on OPC differentiation under oxidative stress and inflammation, we first determined the efficacy of the compounds to promote antioxidant enzyme expression in OPCs. Hereto, primary OPCs were incubated for 24 h with SFN (5 µM), MMF (90 µM) and Protandim (30 µg/mL) or vehicle control. Protandim and SFN significantly enhanced HO-1 protein expression in OPCs (Figure 6). NQO-1 levels were undetectable by Western blotting in OPCs (data not shown). ROS are known to inhibit OPC differentiation [25]. Based on the beneficial effects of Protandim, we next explored the potential of Protandim to promote differentiation of OPCs under oxidative conditions. OPCs were incubated for 24 h with Protandim (30 µg/mL) or vehicle control prior to 5 day exposure with medium containing 0.5% FCS or medium with 10 µM tertbutyl-hydrogen peroxide and 0.5% FCS, but no vehicle control or Protandim. Exposure to 10 µM tertbutyl-hydrogen peroxide resulted in significant and consistent inhibition of OPC maturation, and did not result in significant OPC cell death (data not shown). The ratio of MBP-positive and -negative Olig2-positive cells indicates the percentage of differentiated, MBP-expressing OLs per condition. Protandim treatment slightly increased the percentage of MBP-positive OLs compared to vehicle control under normal conditions (38% ± 3.35% and 31% ± 0.9%, respectively), but this was not significant. Tert-butyl hydrogen peroxide treatment significantly reduced the percentage of MBP-positive OLs compared to untreated vehicle control (25% ± 1.25% and 31% ± 0.9%, respectively). Protandim treatment significantly enhanced the percentage of MBP-positive OLs compared to vehicle control under oxidative stress (34% ± 1.25% and 25% ± 1.75%, respectively) (Figure 7). The percentage of MBP-positive OLs in untreated medium was comparable to that in vehicle control-treated medium.

**Figure 8:**
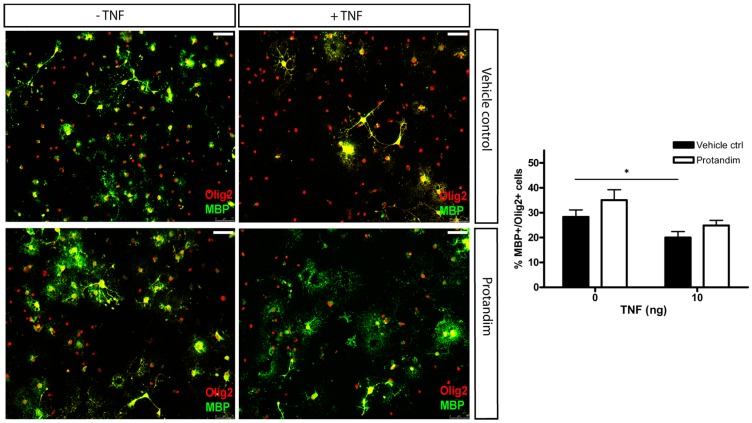
Protandim marginally promotes differentiation of primary rat OPCs in the presence of TNF. After 2 days of de-differentiation, primary rat OPCs were incubated with 30 µg/mL Protandim or vehicle control (EtOH) for 24 h. After removal of medium, cells were subsequently exposed to either control medium or medium with 10 ng/mL TNF for 5 days. MBP and Olig2 expression were assayed by immunocytochemistry. Data are presented as percentage of control and expressed as the mean ± SEM of 3 independent experiments. Statistics reflect student’s *t*-test, one-tailed; * *p* < 0.05.

Inflammatory mediators, such as TNF, are known to inhibit OPC differentiation [30,31]. We incubated OPCs with Protandim (30 µg/mL) or vehicle control and subsequently exposed OPCs to either control medium or medium containing 10 ng/mL TNF in the absence of vehicle control or Protandim for 5 days. This low dosage did not lead to significant OPC cell death (data not shown). TNF significantly decreased the percentage of MBP-positive OLs compared to untreated vehicle control (20% ± 2% and 28% ± 2.8%, respectively). Protandim marginally increased the percentage of MBP-positive oligodendrocytes compared to vehicle control upon TNF treatment (25% ± 2% and 20% ± 2%, respectively), albeit not significantly (Figure 8). The percentage of MBP-positive OLs in untreated medium was comparable to that in vehicle control-treated medium.

## 4. Discussion

In this study, we explored the efficacy of several cytoprotective compounds in protecting OLs against an oxidative and inflammatory insult. We show that the cytoprotective compounds sulforaphane (SFN), monomethyl fumarate (MMF) and Protandim increase the expression of proteins involved in antioxidant protection in OLN-93 cells, whereas Protandim and SFN promote antioxidant enzyme production in mature primary rat OLs and primary rat OPCs. Importantly, SFN and Protandim pre-treatment rescued OLN-93 cells from an oxidative attack. Protandim, but not SFN, significantly enhanced glutathione levels in primary rat OLs and rescued primary rat OLs from an oxidative insult. It is known that pro-inflammatory mediators, such as TNF and ROS inhibit OPC differentiation [25,31]. Here, we show that Protandim, a well-defined combination of five widely studied herbal ingredients and a potent inducer of the Nrf-2 pathway [44], attenuates ROS-induced OPC inhibition.

Early active demyelinating MS lesions are characterized by massive influx of monocyte-derived macrophages and activated microglia. These inflammatory cells produce cytotoxic mediators, such as ROS and inflammatory molecules, including TNF, resulting in OL damage and loss, demyelination and axonal damage [1,2]. To date, evidence is accumulating that compounds boosting endogenous antioxidant production show beneficial effects in various in vitro and in vivo models of neuroinflammation and oxidative stress [33,34,35,36,37,42,43,44,45,59], including the experimental autoimmune encephalomyelitis (EAE) animal model for MS [38,39,60]. In addition, as clinical efficacy of Tecfidera™ in MS was shown to be partly attributable to Nrf2-activation, it suggests that boosting the endogenous antioxidant system in MS has therapeutic potential. Indeed, reports have shown that ROS-induced oxidative damage may play a key role in demyelination in MS [7,8,9,10,11,12] and that Nrf2-driven genes are upregulated in white matter lesions [61].

Notably, all three compounds investigated in our study increased the expression of the cytoprotective proteins HO-1, NQO-1 and p62 in OLN-93 cells. Yet, only SFN and Protandim pre-treatment were able to rescue OLN-93 cells from hydrogen peroxide-induced cell death. This suggests that protein levels of HO-1, NQO-1 and p62 induced by MMF may have been insufficient to prevent ROS-induced cell death. Using primary mature OLs we observed that Protandim treatment resulted in a more pronounced increase in HO-1, NQO-1 and p62 protein expression compared with MMF and SFN. In addition, Protandim treatment significantly increased intracellular glutathione levels, whereas both MMF and SFN did not affect glutathione levels. Glutathione is the most abundant mammalian intracellular thiol-containing antioxidant and represents a key buffer to maintain the cellular redox balance [56,57,58]. Protandim pre-treatment, not MMF or SFN, protected mature primary rat OLs from hydrogen peroxide- and glucose oxidase-induced cell death. These findings indicate that high endogenous levels of glutathione might be essential in protecting OLs against an oxidative attack.

Furthermore, p62 was reported to have neuroprotective effects in SH-SY5Y neuroblastoma cells against hydrogen peroxide-induced cell death [62]. This suggests that Protandim-mediated upregulation of p62 in primary rat OLs may have played a role in their survival against ROS, but this requires further research.

The high metabolic rate, large intracellular iron stores and relatively low levels of endogenous antioxidants make OPCs particularly vulnerable to oxidative and inflammation-driven cell death and injury. The low levels of antioxidant proteins in OPCs may potentially clarify why we could not detect NQO-1 protein levels in primary rat OPCs [14,15,23,24]. Furthermore, ROS are known to hamper OPC differentiation by enhanced expression of genes involved in inhibition of differentiation and decreased expression of genes known to promote OPC differentiation. In our study, ROS also reduced OPC differentiation, which is in line with the report by French and colleagues [25].

We show that Protandim pre-treatment was able to counteract ROS-induced inhibition of OPC differentiation. Interestingly, even under normal conditions we observed a slight increase in the number of mature oligodendrocytes upon exposure to Protandim, suggesting that Protandim, even in the absence of an oxidative insult, might stimulate OPC maturation. Like free radicals, pro-inflammatory cytokines, such as TNF, contribute to OPC cell damage and we observed a reduction in OPC differentiation upon TNF exposure, which was in line with previous reports [30,31]. In contrast to the beneficial effects observed under oxidative conditions, Protandim treatment did not attenuate TNF-mediated block of OPC differentiation.

Athough Protandim showed high potency in our rat OL cultures, and demonstrated efficacy in animal models of cardiovascular disease [43,63], little is known about the pharmacodynamic and pharmacokinetic properties. Protandim contains five herbal ingredients, including ashwagandha, bacopa extract, green tea extract, silymarin, and curcumin, and several studies have shown that Protandim is able to potently activate Nrf2-driven gene expression [44,45]. The therapeutic properties of all five compounds have been reported previously [64,65,66], of which curcumin has been the most thoroughly investigated [67,68,69]. Further research into the mode of action of Protandim is therefore required in order to determine if the compound would be efficacious in animal models for MS and MS patients.

## 5. Conclusions

Taken together, our findings indicate that Protandim effectively prevented ROS-driven oligodendrocyte cell death and promoted OPC differentiation under oxidative conditions. Our in vitro data warrants future research to further explore the therapeutic efficacy of Protandim in experimental MS animal models.

## Supplementary Materials

The following are available online at www.mdpi.com/2076-3921/5/3/30/s1. Table S1. Antibody characterization.
