# Supplementary material for: Protandim Protects Oligodendrocytes against an Oxidative Insult
Source: Antioxidants (Basel). 2016 Sep 7;5(3):30. doi: 10.3390/antiox5030030 (PMC5039579; doi:10.3390/antiox5030030)
Supplement: Supplementary file 1 [file antioxidants-05-00030-s001.pdf]

Supplementary Materials: Protandim Protects Oligodendrocytes against an Oxidative Insult

Jamie L. Lim, Susanne M. A. van der Pol, Wia Baron, Joe M. McCord, Helga E. de Vries, and Jack van Horssen

Table S1. Antibody characterization.

Antibody	Immunogen	Source	Dilution
Anti-MBP	Bovine MBP	Abd serotec, catalog no. MCA409S, RRID:AB_325004, rat, monoclonal	1:200 (fluorescence/WB)
Anti-Olig2	Recombinant mouse Olig-2	Chemicon, catalog no. AB9610, RRID:AB_570666, rabbit, polyclonal	1:200 (fluorescence)
Anti-HO-1	Native rat liver HO-1 (Hsp32) protein	Enzo Life Sciences, catalog no. ADI-OSA-150F, RRID:AB_1505620, rabbit, polyclonal	1:1000 (WB)
Anti-NQO-1	Recombinant full-length protein of human NQO-1	Abcam, catalog no. ab28947, RRID:AB_881738, mouse, monoclonal	1:1000 (WB)
Anti-p62/SQSTM1	Recombinant full length protein, corresponding to amino acids 1-441 of Human SQSTM1/p62	Abcam, catalog no. ab56416, RRID:AB_945626, mouse, monoclonal	1:1000 (WB)
Anti-β-actin	Slightly modified ß-cytoplasmic actin N-terminal peptide, Ac-Asp-Asp-Asp-Ile-Ala-Ala-Leu-Val-Ile-Asp-Asn-Gly-Ser-Gly-Lys, conjugated to KLH	Sigma Aldrich, catalog no. A5441, RRID:AB_476744, mouse, monoclonal	1:5000 (WB)
Anti-rat 488	IgG (H+L) secondary antibody, Alexa-Fluor 488 conjugate	Thermo Fischer Scientific, catalog no. A-11006, RRID:AB_10561520, goat, polyclonal	1:200 (fluorescence)
Anti-rabbit 555	IgG (H1L) secondary antibody, Alexa-Fluor 555 conjugate	Thermo Fischer Scientific, catalog no. A-21428, RRID:AB_2535849, goat, polyclonal	1:200 (fluorescence)

© 2016 by the authors; licensee MDPI, Basel, Switzerland. This article is an open access article distributed under the terms and conditions of the Creative Commons by Attribution (CC-BY) license (http://creativecommons.org/licenses/by/4.0/).
